# Lipopolysaccharide-Induced Lung Injury Is Independent of Serum Vitamin D Concentration

**DOI:** 10.1371/journal.pone.0049076

**Published:** 2012-11-19

**Authors:** Lindy S. Klaff, Sean E. Gill, Brent E. Wisse, Kristen Mittelsteadt, Gustavo Matute-Bello, Peter Chen, William A. Altemeier

**Affiliations:** 1 Center for Lung Biology, Department of Medicine, University of Washington, Seattle, Washington, United States of America; 2 Diabetes and Obesity Center of Excellence, Department of Medicine, University of Washington, Seattle, Washington, United States of America; Leiden University Medical Center, Netherlands

## Abstract

Vitamin D deficiency is increasing in incidence around the world. Vitamin D, a fat-soluble vitamin, has documented effects on the innate and adaptive immune system, including macrophage and T regulatory (Treg) cell function. Since Treg cells are important in acute lung injury resolution, we hypothesized that vitamin D deficiency increases the severity of injury and delays injury resolution in lipopolysaccharide (LPS) induced acute lung injury. Vitamin D deficient mice were generated, using C57BL/6 mice, through diet modification and limited exposure to ultraviolet light. At 8 weeks of age, vitamin D deficient and sufficient mice received 2.5 g/kg of LPS or saline intratracheal. At 1 day, 3 days and 10 days, mice were anesthetized and lung elastance measured. Mice were euthanized and bronchoalveolar lavage fluid, lungs and serum were collected. *Ex vivo* neutrophil chemotaxis was evaluated, using neutrophils from vitamin D sufficient and deficient mice exposed to the chemoattractants, KC/CXCL1 and C5a, and to bronchoalveolar lavage fluid from LPS-exposed mice. We found no difference in the degree of lung injury. Leukocytes were mildly decreased in the bronchoalveolar fluid of vitamin D deficient mice at 1 day. *Ex-vivo*, neutrophils from vitamin D deficient mice showed impaired chemotaxis to KC but not to C5a. Vitamin D deficiency modestly impairs neutrophil chemotaxis; however, it does not affect lung injury or its resolution in an LPS model of acute lung injury.

## Introduction

Vitamin D deficiency is common in the United States (US) and worldwide, and serum levels in the US are lower now than they were 10 years ago [1], [2], [3]. Its role in the prevention of rickets was published in 1922 in a paper by Hess [4] and its importance in musculoskeletal health has been well known for decades. Vitamin D_3_ (cholecalciferol) either is made in the skin when 7-dehydrocholesterol reacts with ultraviolet (UV) light or is acquired through dietary intake. Vitamin D_3_ is further hydrolyzed by the liver to create 25-hydroxyvitamin D_3_ (25[OH]D_3_), which reflects the body's store of vitamin D. Additional conversion of 25[OH]D_3_ to the more biologically active 1α,25-hydroxyvitamin D_3_ (1α,25[OH]D_3_) occurs primarily in the kidney [5], but, is also observed in other cell types such as macrophages [6], respiratory epithelial cells [7], and others [8], [9].

The potential role of vitamin D on immune function has been explored in the last decade. Autoimmune diseases such as type 1 diabetes [10], multiple sclerosis [11] and asthma [12], [13] have been associated with vitamin D deficiency. This is largely thought to be due to the effects of vitamin D on T-regulatory (Treg) cell function and vitamin D's ability to stimulate Treg populations and enhance tolerance [14], [15], [16]. The effects of vitamin D on macrophage function have been well studied in *Mycobacterium tuberculosis (MTb)*, and clinical tuberculosis is associated with vitamin D deficiency [17], [18]. Macrophage release of cathelicidin, an antimicrobial peptide required for efficient macrophage killing of *MTb*, in response to Toll-like receptor activation requires co-activation of the vitamin D receptor [19], [20]. Neutrophils also express the vitamin D receptor [21]; however, beyond the fact that patients with rickets have impaired neutrophil chemotaxis [22], little else is known about vitamin D regulation of neutrophil function.

These data suggest that vitamin D levels may modulate pulmonary inflammatory responses and/or resolution of inflammation in response to infectious stimuli. Multiple studies have identified a correlation between severe vitamin D deficiency in children and lower respiratory tract infections such as influenza [23], [24], [25], [26], [27]. Recent studies have also correlated low cord-blood vitamin D levels with infant wheezing [28] and risk for RSV infection [29].

Relatively few animal studies to date have evaluated the relationship between vitamin D and acute lung injury (ALI), and those that have been published examine the effect of vitamin D supplementation above normal dietary intake [30], [31], [32]. ALI is often associated with Toll-like receptor activation by either microbial products or damage-associated molecular patterns (DAMPs) and involves an acute inflammatory stage, which is predominantly neutrophilic, followed by resolution and repair [33]. Lung injury resolution is dependent on many host factors, including transition of pro- to anti-inflammatory cytokines and neutrophil clearance [33]. Using a murine model of lipopolysaccharide (LPS) -induced injury, a critical role for Tregs has recently been recognized in resolution of lung injury [34].

We hypothesized that vitamin D level would influence LPS-induced lung inflammation and injury, and its resolution. To test this hypothesis, we developed a murine model of vitamin D deficiency through diet modification to compare the temporal pattern of inflammation and resolution in response to LPS exposure. We found that vitamin D alters neutrophil chemotaxis *ex vivo* but does not significantly affect the extent of LPS-induced lung injury or its resolution *in vivo*.

## Materials and Methods

### Generation and characterization of vitamin D sufficient (VDS) and vitamin D deficient (VDD) mice

The University of Washington Office of Animal Welfare approved these experiments. Wild-type C57BL/6 mice were purchased from Jackson laboratory (Bar Harbor, Maine) and bred in a modified specific pathogen free facility. When female breeder mice were visibly pregnant, they were either placed in vitamin D sufficient or deficient housing. Vitamin D deficient housing included a diet that was absent of vitamin D and contained 1% calcium (Harlan, Madison, WI). The cages were wrapped in ultraviolet blocking film (UV Process Supply, Chicago, IL) to prevent any cutaneous conversion of vitamin D. The vitamin D sufficient mice were exposed to normal vivarium lighting and received an identical diet to the deficient mice with an additional 1000 IU/kg of cholecalciferol (Harlan, Madison, WI). Offspring were maintained under the same conditions as their mother.

Serum was obtained from 6-week old mice by cardiac puncture and stored at −80°C. The serum was analyzed for 25[OH]D_3_ by liquid chromatography-tandem mass spectrometry (Waters, Milford, MA). Serum from 8-week old mice was analyzed for alkaline phosphatase, phosphate and calcium by immunoassay (Beckman Coulter, Brea, CA). Complete blood cell count and differential was measured from whole blood (Phoenix Central Laboratory, Seattle, WA).

Body composition was measured using a quantitative magnetic resonance (QMR) method using the Echo MRI™ 3-in-1 Animal Tissue Composition Analyzer (Echo Medical Systems, Houston, TX). Measurements were made in triplicate for each mouse.

Food consumption was estimated by weighing food every 2–3 days over a two-week period in 6 to 8 week-old mice prior to LPS or PBS instillation. Weight of food eaten was divided by number of mice per cage per day.

### LPS preparation

A stock solution of LPS derived from *Escherichia coli* O55:B5 (Sigma-Aldrich, St. Louis, MO) in phosphate buffered saline (PBS) was stored at −20°C. Immediately before each experiment, an aliquot of LPS was thawed, sonicated and resuspended in PBS to a working concentration of 1 mg/mL.

### Experimental protocol

Each mouse was anesthetized with 5% isoflurane and suspended by its front teeth at a 60° angle. After extending the jaw and tongue, 2.5 µg/g body weight of LPS or an equal volume of sterile PBS was deposited in the oropharynx with a pipette. Aspiration of the liquid was visually confirmed, and the mouse returned to its cage. Female and male mice were equally distributed among the groups.

1, 3, or 10 days after LPS or PBS instillation, mice were anesthetized with 5% isoflurane and underwent tracheotomy and intubation with a blunt tip 18-gauge needle (BD Bioscience, San Jose, CA), which was secured with 3-0 silk suture (Look, Reading, PA). Mice were connected to a ventilator (Flexivent, Scireq, Montreal, QC) and received pancuronium (Hospira, Lake Forest, IL) at 1 mg/kg body weight. The mice were ventilated with a tidal volume of 10 mL/kg body weight at a respiratory rate of 150 breaths/minute, an end-expiratory pressure of 3 cmH_2_O and 0.21 FiO_2_ while receiving 2% isoflurane as previously described [35]. After a 5-min stabilization period, the lungs were twice inflated to an end-inspiratory pressure of 30 cmH_2_O. Multi-frequency forced oscillation waveform maneuvers were repeated 12 times to determine lung elastance (H), using the constant phase model as previously reported [36], [37].

After physiological data were collected, mice were euthanized, a median sternotomy was performed and the left lung lobe isolated, removed and weighed.

### BAL fluid analysis

BAL of the right lung lobes was performed with 3 aliquots of 0.5 mL PBS containing 0.6 mM EDTA. BAL fluid was centrifuged at 800 *g* for 10 minutes. The supernatant was removed and stored at −80°C for future analysis. The cell pellet was resuspended in 200 µL of PBS with 0.6 mM EDTA and cell count was performed using the Easy Count System (Immunicon, Huntingdon Valley, PA). Differential cell count was determined by cytocentrifugation and Wright-Giemsa staining.

BAL fluid cytokine concentrations were determined for MIP-2/CXCL2, mouse growth-related oncogene homologue (KC/CXCL1), IL6, and TNFα by ELISA (R&D Systems, Minneapolis, MN). Total protein concentrations were determined by BCA assay (Pierce, Rockford, IL), and IgM levels were measured by ELISA (Bethyl laboratories, Montgomery, TX).

### Histology and Immunohistochemistry

For three mice per group, the right lung lobes were removed after BAL and fixed at 15 cm H_2_O with 4% paraformaldehyde. After fixation, the lungs were embedded in paraffin. For each tissue block, four 4-µm sections were prepared after cutting ∼100 µm into the lung. One section was stained with hematoxylin and eosin, and the remaining sections were reserved for immunohistochemistry (IHC).

All IHC was performed, using the Leica Bond Automated Immunostainer and all reagents, unless otherwise stated were purchased from Leica Microsystems, Inc. (Buffalo Grove, IL). Following deparaffinization and antigen retrieval with HIER 2 solution, slides were stained for macrophages or neutrophils, using α-F4/80 (clone BM8, 1∶200 dilution, Invitrogen, Grand Island, NY) or α-Ly6B (clone 7/4, 1∶10,000 dilution, AbD Serotec, Raleigh, NC), respectively. All slides were then incubated with rabbit-anti-rat secondary antibody (1∶300 dilution, Vector Laboratories, Burlingame, CA), stained with DAB, and counterstained with hematoxylin. Rat IgG 2b isotype control (1∶500 dilution, BD Biosciences, San Jose, CA) sections were included for all slides.

### Measurement of neutrophil chemotaxis and CXCR2 Expression ex vivo

Vitamin D sufficient and deficient mice were euthanized by exposure to isoflurane followed by exsanguination. The femurs and tibia of both hind legs were dissected free, and neutrophils were isolated from the bone marrow as previously described [38].

To evaluate chemotaxis, isolated neutrophils were labeled with calcein-AM (5 mg/mL; Molecular Probes, Eugene, OR) for 30 minutes at 37°C, washed two times in phosphate buffered saline (PBS) and resuspended at a concentration of 1×10^6^/mL. Chemotaxis was assessed using the 96-well Neuro Probe ChemoTx® Disposable Chemotaxis system (Neuro Probe. Gaithersburg, MD). Individual wells were filled with BAL fluid from Vitamin D sufficient and deficient mice 1 day after LPS instillation, or with various concentrations of KC (PeproTech, Rocky Hill, NJ), C5a (R&D Systems), or PBS. A polycarbonate filter (8 µm pores) with a hydrophobic ring around the area over each well was placed on the 96-well plate and calcein-labeled neutrophils were added to each ring. The chemotaxis chamber, consisting of the polycarbonate filter and 96-well plate, was incubated for 30 min at 37°C in 5% CO_2_ and then non-migrating neutrophils were removed from the upper side of the filter. The chemotaxis chamber was placed in a multi-well fluorescent plate reader (Synergy 4, BioTek, Winooski, VT) and the migrated cells were measured using the calcein fluorescence signal (excitation - 485 nm, emission - 530 nm). Neutrophil migration was expressed as a percent of the total number of neutrophils that were placed on the topside of the filter after normalization for random migration (% Total).

To evaluate CXCR2 expression, neutrophils isolated from the bone marrow of vitamin D –sufficient and –deficient mice were washed in FACS buffer (PBS+0.5% BSA), blocked with rat anti-mouse CD16/32 (FC Block, BDBiosciences), incubated with either PE-conjugated rat anti-mouse CXCR2 or rat isotype control IgG_2A_ antibody (R&D systems), washed, and analyzed in a Guava benchtop flow cytometer (Millipore, Billerica, MA). Neutrophils were identified on forward and side scatter plots while gating out debris. CXCR2 positive cells were defined as having fluorescent intensity greater than 95% of cells labeled with isotype control antibody.

### Statistical analysis

BAL fluid measurements, including cell counts, were analyzed by 2-way ANOVA with Bonferroni post-test analysis. Two group comparisons were performed using the Student's t-test. Lung elastance over time was compared between two groups by repeated measures ANOVA. Neutrophil chemotaxis data were analyzed by linear regression. All analyses were performed with Prism 5 (Graphpad software, La Jolla, CA). All data are presented as mean ± standard deviation unless otherwise noted.

## Results

### Baseline characteristics of vitamin D sufficient and deficient mice

Vitamin D deficiency (VDD) and sufficiency (VDS) was confirmed in 6-week old mice. All VDD mice had serum levels of 25[OH]D_3_ below the lower limit of detection as compared with VDS mice, which had 25[OH]D_3_ levels of 14.6±2.6 ng/mL. Calcium and phosphate levels were not significantly different between groups. Alkaline phosphatase was significantly higher in the VDD group as compared with the VDS group, likely reflecting increased bone resorption as a response to secondary hyperparathyroidism, resulting from vitamin D deficiency. Additionally, blood total leukocyte and neutrophil counts were similar between groups (Table 1).

**Table 1:** Serum and whole blood measurements.

	VDS^a^ Mean (SD)	VDD^b^ Mean (SD)	p-value
25(OH)Vitamin D3 (ng/mL)	14.6 (2.6)	not detected	0.0006
Calcium (mg/dL)	8.8 (0.8)	8.6 (0.5)	0.15
Phosphate (mg/dL)	7.6 (1.2)	7.4 (1.1)	0.81
Alkaline phosphatase (U/L)	105.2 (14.3)	150.7 (35.7)	0.03
Leukocyte (×10^3^/µL)	4.35 (1.5)	4.21 (1.7)	0.88
Absolute neutrophil count (×10^3^/µL)	0.395 (0.183)	0.375 (0.253)	0.88

a vitamin D sufficient mice;

b vitamin D deficient mice.

**Figure 1:**
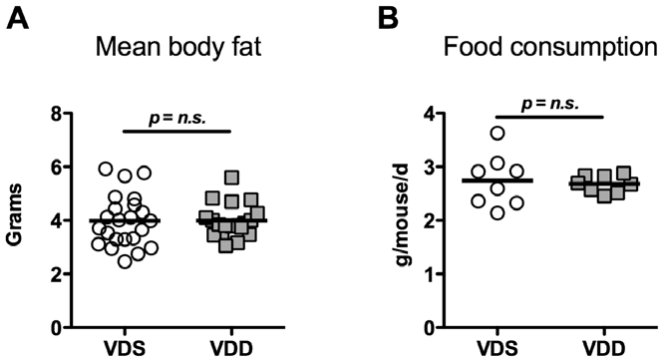
Body composition and food intake. (**A**) Mean body fat in vitamin D sufficient (VDS, n = 23) and vitamin D deficient (VDD, n = 19) mice of both genders as determined by quantitative magnetic resonance. (**B**) Food consumption by VDS and VDD mice averaged over two weeks as determined by measuring food weight loss per cage divided by number of mice per cage and the number of days between measurements. n = 8 cages/genotype.

VDS and VDD mice were similar in size at 8 weeks. Sufficient versus deficient male mice weighed (23.6±2.2 g vs. 23.5±1.4 g). Female sufficient versus deficient mice weighed (18.9±1.1 g vs. 18.5±1.6 g). We further investigated body composition of the mice through quantitative magnetic resonance. The baseline mean body fat, percent body fat and mean body lean was the same in both groups (Figure 1A). Mouse food intake was also similar between VDS and VDD groups (2.74±0.49 g/mouse/day vs. 2.68±0.16 g/mouse/day) (Figure 1B).

Lung injury and inflammation were measured by a variety of parameters at days 1, 3, and 10 after instillation with either PBS or LPS. There were no significant differences observed between PBS instilled VDS mice and VDD mice at any of the times assessed for any of the measured parameters.

### Lung injury and inflammation 1 day after LPS

**Figure 2:**
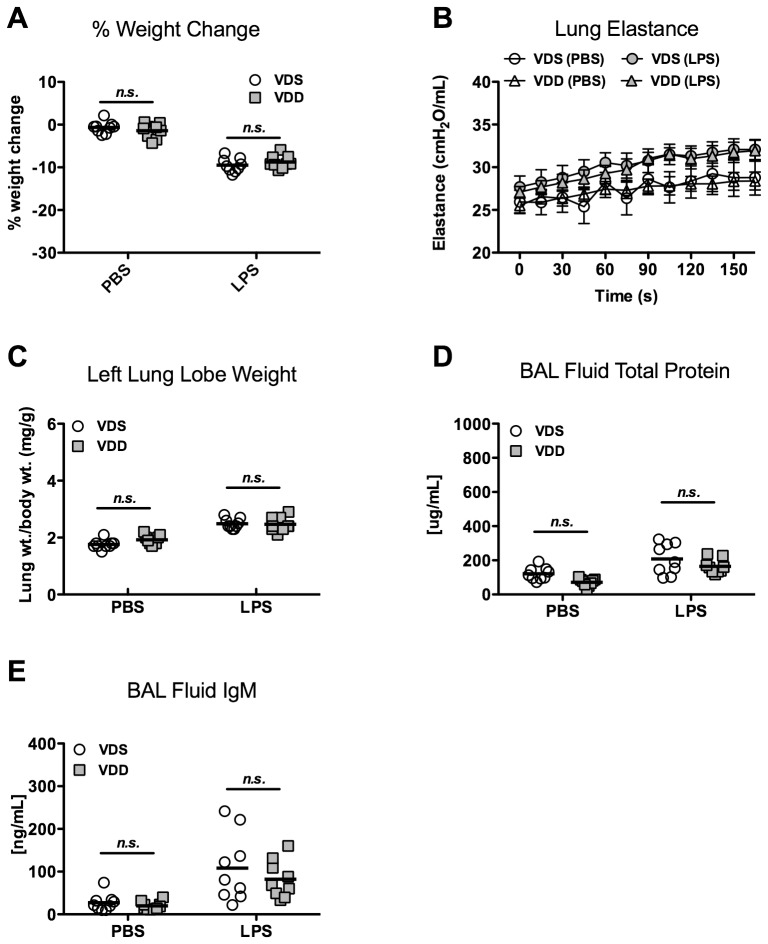
Lung injury 1 day after LPS instillation. Vitamin D-sufficient (VDS) or –deficient (VDD) treated with either LPS or PBS. N = 7 to 9 per group. (**A**) Percent body weight change; (**B**) Lung elastance (H) over time following lung recruitment (mean ± SEM); (**C**) Left lung lobe weight (wt) normalized to day 0 body weight; (**D**) Total protein concentration of bronchoalveolar lavage (BAL) fluid; (**E**) IgM concentration of BAL fluid.

LPS treatment caused significant weight loss (p<0.001) in both VDS and VDD groups as compared to PBS controls. However, percent weight change was not significantly different between VDS and VDD mice 1 day post-LPS treatment (−9.5±1.6% and −8.8±1.5%, respectively) (Figure 2A). Lung elastance was overall significantly higher after LPS treatment as compared with PBS treatment. However, following LPS treatment, maximum lung elastance was not different between the VDS and VDD mice (32.4±3.5 vs. 31.9±3.0 cmH_2_O/mL, respectively) (Figure 2B). Lung water was assessed by left lung lobe weight normalized to day 0 body weight to correct for variations in lung size. LPS treatment resulted in greater normalized lung weights overall (p<0.001), but no difference was found between VDS and VDD mice 1 day after LPS treatment (2.49±0.18 vs. 2.47±0.25 mg/g, respectively) (Figure 2C). Similarly, LPS treatment caused significantly higher BAL concentrations of both total protein (p<0.001) and IgM (p<0.001), indicating increased alveolar-capillary permeability. No significant differences were observed between VDS and VDD mice after LPS treatment groups in total protein concentration (207.8±89.3 vs. 164.2±41.9 µg/mL) or IgM concentration (108.2±79.2 vs. 82.1±43.6 ng/mL)(Figure 2D, 2E).

**Figure 3:**
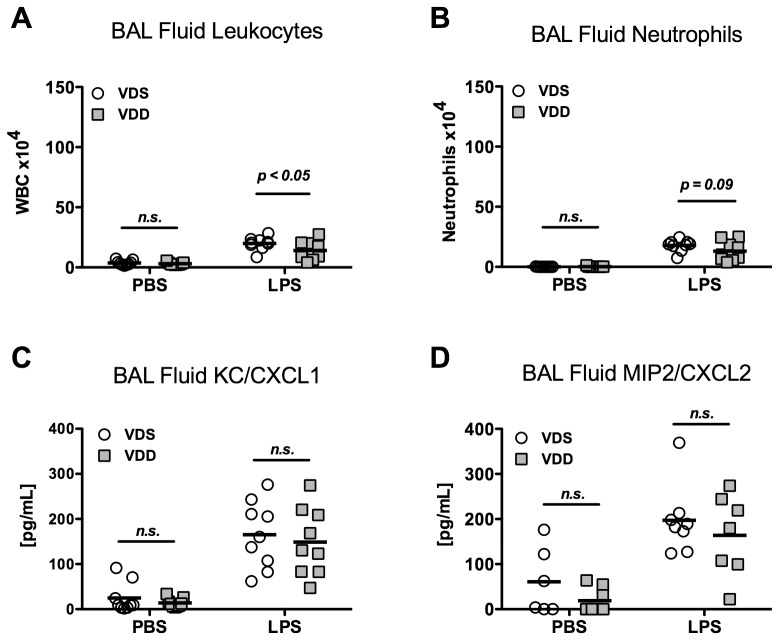
Lung inflammation 1 day after either LPS instillation. Vitamin D-sufficient (VDS) or –deficient (VDD) treated with either LPS or PBS. N = 7 to 9 per group. (**A**) Bronchoalveolar lavage (BAL) fluid total cell count, p<0.05 comparing VDS and VDD mice after LPS instillation; (**B**) BAL fluid neutrophil count, p = 0.09 comparing VDS and VDD mice after LPS; (**C**) BAL fluid KC/CXCL1 concentration; (**D**) BAL fluid MIP2/CXCL2 concentration.

LPS exposure was associated with significantly more leukocytes in BAL fluid (p<0.001). In contrast to measurements of lung mechanics and permeability, total BAL fluid leukocyte count was modestly higher after LPS treatment in VDS versus VDD mice (2.0±0.54×10^5^ vs. 1.4±0.78×10^5^ cells, p<0.05, Figure 3A). A similar trend was observed in BAL fluid neutrophils after LPS treatment, although the difference between VDS and VDD did not reach statistical significance (1.8±0.49×10^5^ vs.1.3±0.81×10^5^ cells, p = 0.09) (Figure 3B). BAL fluid macrophages/monocytes were similar after LPS in both VDS and VDD mice (0.20±0.11×10^5^, vs. 0.16±0.07×10^5^, p = n.s.). Minimal BAL fluid lymphocytes were observed in both VDS and VDD mice treated with LPS (0.04±0.06×10^4^ vs. 0.05±0.06×10^4^, p = n.s.). Although BAL fluid concentrations of the neutrophil chemokines, KC/CXCL1 and MIP-2/CXCL2, were increased after LPS, no differences were found between VDS and VDD mice (165.0±74.0 vs. 148.8±75.0 pg/mL (KC/CXCL1) and 197.0±76.5 vs. 163.8±90.6 pg/mL (MIP2/CXCL2)) (Figure 3C, 3D). Additionally, levels of the inflammatory cytokines TNFα and IL6 were similar between VDS and VDD mice within PBS and LPS treated groups (data not shown).

**Figure 4:**
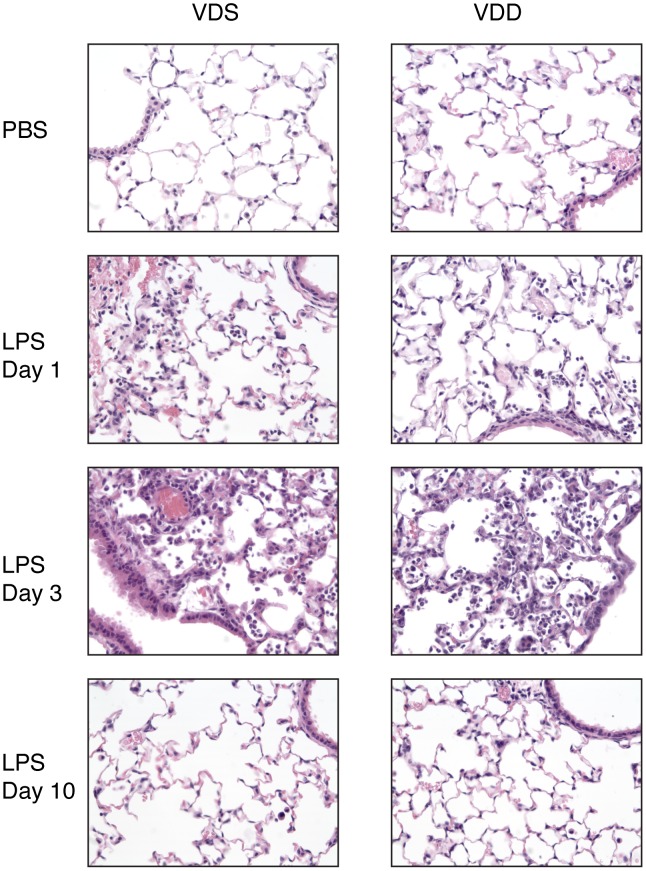
Representative histology sections. 400× magnfication of hematoxylin and eosin stained sections of right lung lobes collected from vitamin D sufficient (VDS) and deficient (VDD) mice after exposure to PBS or to LPS for varying lengths of time.

Lung histological sections demonstrate mildly increased cellularity in both VDS and VDD mice one day after LPS exposure as compared to PBS-exposed animals (Figure 4). At higher magnification, cell morphology of most of the intra-alveolar cells is consistent with polymorphonuclear cells. IHC confirms a predominantly Ly6B positive cell population with no clear differences between VDS and VDD mice (data not shown).

### Lung injury and inflammation 3 days after LPS

**Figure 5:**
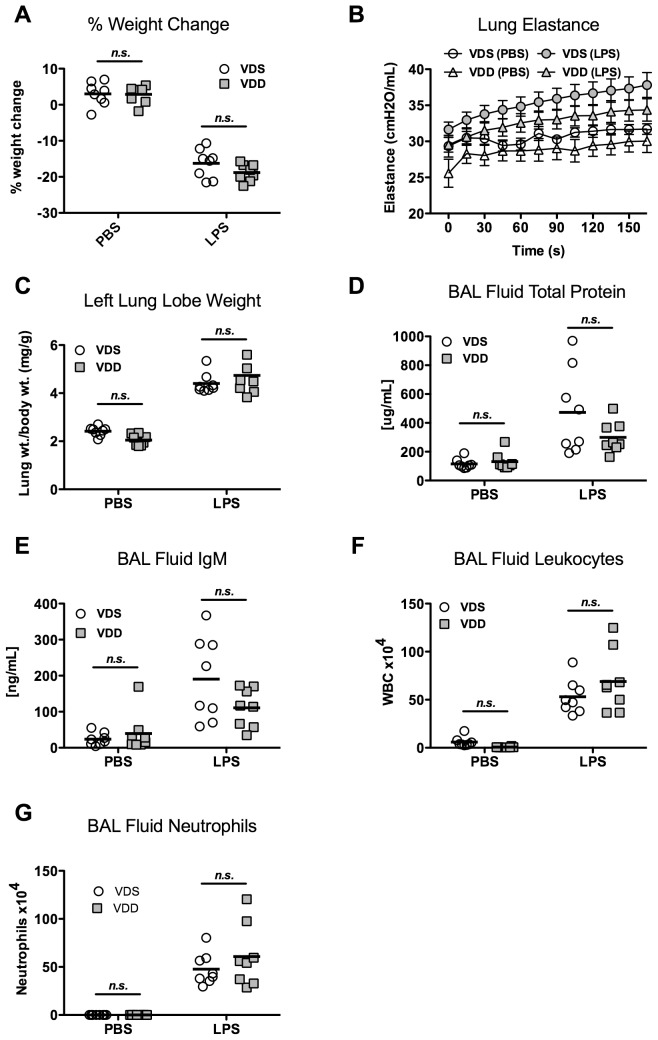
Lung injury and inflammation 3 days after LPS instillation. Vitamin D-sufficient (VDS) or –deficient (VDD) treated with either LPS or PBS. N = 8 per group. (**A**) Percent body weight change; (**B**) Lung elastance (H) over time following lung recruitment (mean ± SEM); (**C**) Left lung lobe weight (wt) normalized to day 0 body weight; (**D**) Total protein concentration of bronchoalveolar lavage (BAL) fluid; (**E**) IgM concentration of BAL fluid; (**F**) BAL fluid total cell count; (**G**) BAL neutrophil count.

Percent weight change 3 days after LPS administration was similar between VDS and VDD mice (16.3±4.0% vs. 18.8±2.2%) (Figure 5A). Day 3 was the nadir of weight loss, and no mice died spontaneously. Lung injury and inflammation were comparable between VDS and VDD mice 3 days after intratracheal LPS. Lung elastance was elevated after LPS treatment as compared with PBS treatment but there was no significant difference between the VDS and VDD mice (37.9±4.8 vs. 34.5±4.0 cmH_2_O/mL) (Figure 5B).

Permeability was not different between VDS and VDD mice 3 days after LPS. Normalized lung weights were similar in VDS and VDD mice (4.40±0.42 vs. 4.74±0.80 mg/g) (Figure 5C). BAL fluid total protein (473.3±295.0 vs. 299.6±106.9 µg/mL) and IgM (190.4±116.4 vs. 111.0±53.5 ng/mL) were similar between VDS and VDD mice after LPS (Figure 5D, 5E).

By day 3 after intratracheal LPS, inflammation was similar in VDS and VDD mice with no difference in total leukocytes (5.3±1.7×10^5^ vs. 6.9±3.1×10^5^ cells, Figure 5F), neutrophils (4.8±1.6×vs. 6.1±3.3×10^5^ cells, Figure 5G), macrophages/monocytes (0.53±0.21×10^5^ vs. 0.84±0.37×10^5^ cells), or lymphocytes (0.03±0.09×10^4^ vs. 0.02±0.06×10^4^ cells) in BAL fluid.

BAL fluid cytokine levels of KC/CXCL1, MIP2/CXCL2, TNFα and IL6 were not significantly different between VDD and VDS mice 3 days after LPS administration (data not shown).

By histology, cellularity was greatest 3 days after LPS and similar in both VDS and VDD mice (Figure 4). Examination at higher magnification and with IHC confirms a predominantly polymorphonuclear cell infiltrate (data not shown).

### Lung inflammation and injury 10 days after LPS

**Figure 6:**
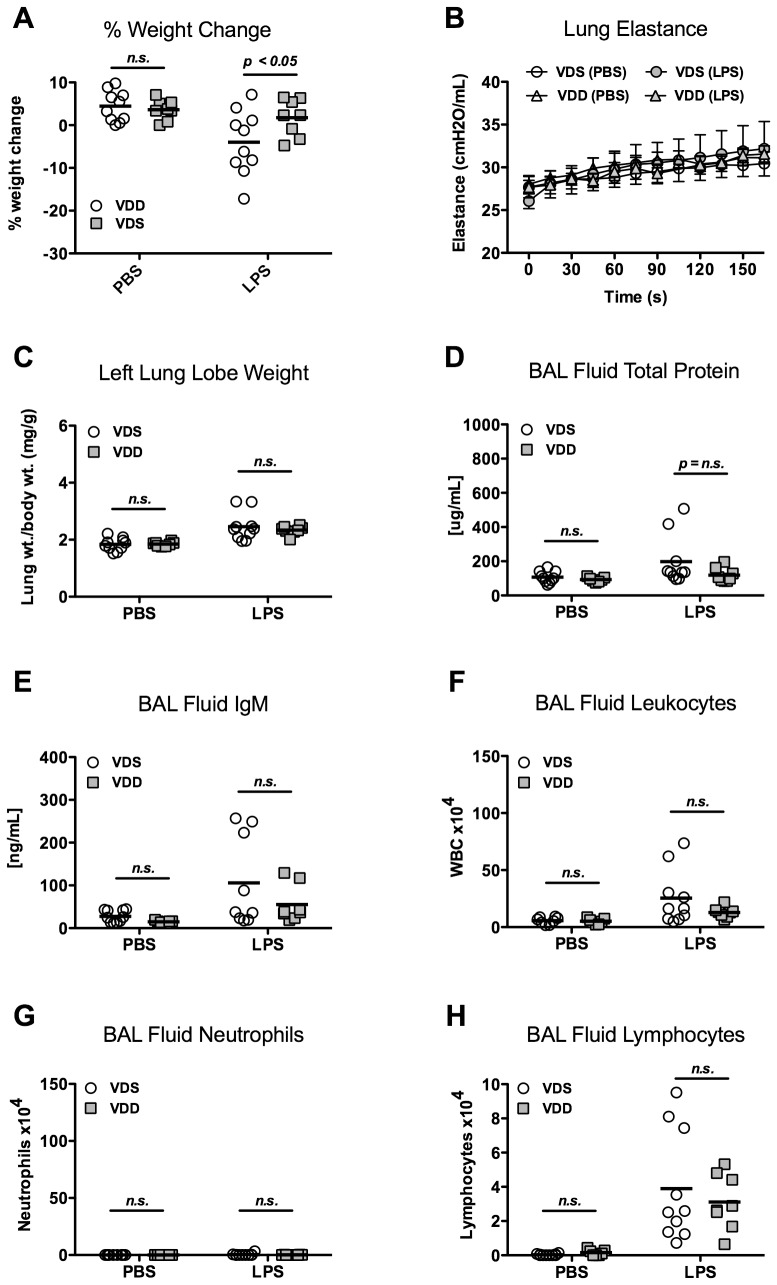
Lung injury and inflammation 10 days after LPS instillation. Vitamin D-sufficient (VDS) or –deficient (VDD) were treated with either LPS or PBS. N = 8 to 10/group. (**A**) Percent body weight change, p<0.05 comparing VDS and VDD mice after LPS; (**B**) Lung elastance (H) over time following lung recruitment (mean ± SEM); (**C**) Left lung lobe weight (wt) normalized to day 0 body weight; (**D**) Total protein concentration of bronchoalveolar lavage (BAL) fluid; (**E**) IgM concentration of BAL fluid; (**F**) BAL fluid total cell count; (**G**) BAL neutrophil count.

To investigate the effect of vitamin D deficiency on resolution of lung injury, we evaluated mice at day 10 after LPS or PBS instillation [34]. Weights of both VDS mice and VDD mice had returned to near baseline by day 10 although there was a small but significant difference in weight change between VDS and VDD mice (−4.0±7.5% vs. 1.8±4.3%, p<0.05) (Figure 6A).

Lung inflammation and injury had resolved to a similar degree in both VDS and VDD mice by day 10 following LPS instillation. Lung elastance after LPS was similar between VDS and VDD mice (32.4±8.9 vs. 31.7±2.2 cmH_2_O/mL) (Figure 6B). There was no difference in lung permeability between VDS and VDD mice as determined by the normalized lung weight (2.46±0.50 vs. 2.34±0.15 mg/g), BAL fluid total protein concentration (198.0±143.9 vs. 119.0±40.9 µg/mL), and BAL fluid IgM concentration (105.7±105.5 vs. 55.1±42.9 ng/mL) (Figures 6C, 6D, 6E). Total BAL fluid leukocyte counts (2.54±2.40×10^5^ vs. 1.28±0.45×10^5^ cells, Figure 6F), BAL fluid neutrophils (0.05±0.10 vs. 0.01±0.01×10^5^ cells, Figure 6G) and BAL fluid macrophages/monocytes (1.18±0.71×10^5^ vs. 0.96±0.36×10^5^ cells) were all statistically similar between LPS-treated VDS and VDD mice. BAL fluid lymphocytes were significantly increased 10 days after LPS treatment but were not different between VDS and VDD mice (3.90±3.21×10^4^ vs. 3.11±1.61×10^4^, Figure 6H).

By day 10, the majority of inflammatory infiltrate had resolved in both VDS and VDD mice. No histological differences were appreciated between VDS and VDD mice (Figure 4).

### Neutrophil chemotaxis

**Figure 7:**
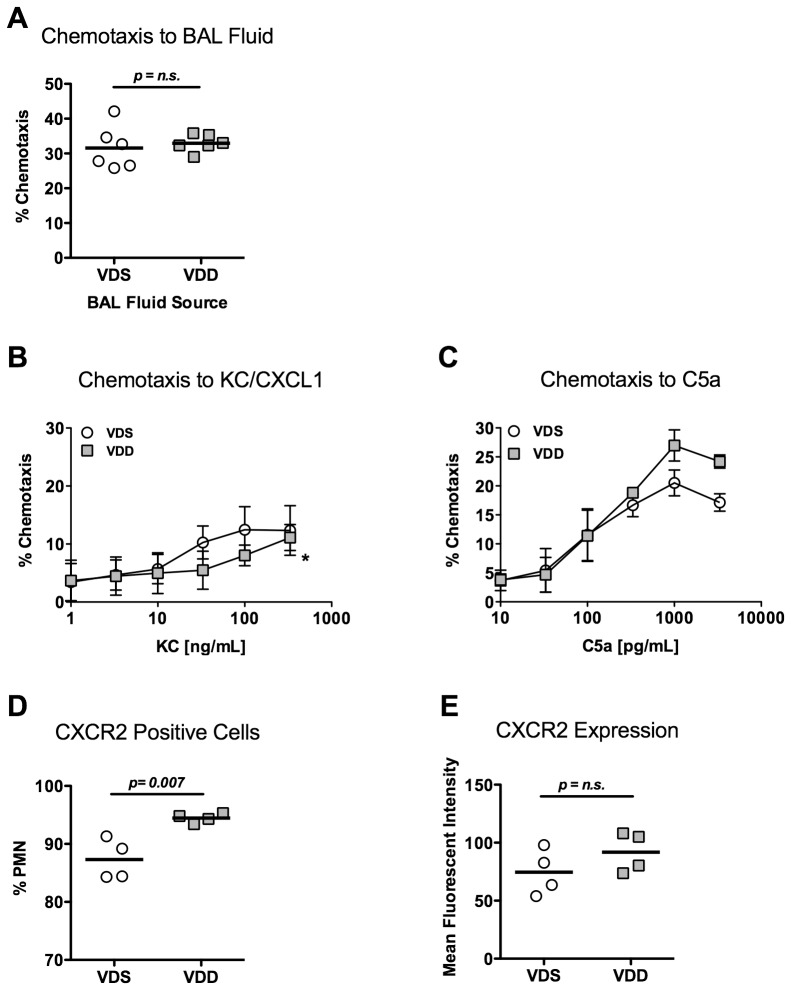
Neutrophil chemotaxis. Neutrophils were isolated from vitamin D-sufficient (VDS) or –deficient (VDD) mouse bone marrow for chemotaxis measurement and flow cytometry. (**A**) VDS neutrophil chemotaxis towards BAL fluid collected from VDS and VDD mice 1 day after LPS stimulation; Chemotaxis of neutrophils isolated from VDS and VDD mice towards varying concentrations of (**B**) KC/CXCL1 (N = 5 per group) and (**C**) complement C5a (N = 3 per group); (**D**) Percentage of CXCR2 positive cells in neutrophils isolated from bone marrow of VDS and VDD mice (N = 4 per group); (**E**) Expression of CXCR2 as determined by mean fluorescent intensity measured with flow cytometry of CXCR2+ cells from bone marrow of VDS and VDD mice (N = 4 per group).

Because of the trend toward lower BAL fluid neutrophil counts 1 day after LPS exposure in VDD mice and because of a prior report that neutrophil chemotaxis is reduced in patients with rickets [22], we measured the chemotactic potential of BAL fluid from LPS exposed VDS and VDD mice on bone marrow-derived neutrophils collected from VDS mice. Chemoattractant potential of BAL fluid from VDD mice was not significantly different from that of BAL fluid from VDS mice (Figure 7A).

Neutrophil chemotaxis was further examined through isolation of neutrophils from VDS and VDD mice and exposure to different chemoattractants. We found that VDD neutrophils had decreased chemotaxis towards KC/CXCL1 as compared with VDS neutrophils (Figure 7B). In contrast, no significant difference in chemotaxis was observed towards C5a (Figure 7C). Because these data suggested that neutrophils from vitamin D deficient mice had impaired chemotaxis to selected chemoattractants, we assessed surface expression of CXCR2, the receptor for KC/CXCL1, by flow cytometry, on bone marrow-derived neutrophils isolated from VDS and VDD mice. The number of CXCR2+ cells was similar between the two groups, although there was a significantly lower percentage of CXCR2 positive cells isolated from VDS mice as compared to VDD mice (87.3±3.5% vs. 94.5±0.8%, p = 0.007, Figure 7D). There was no significant difference in the mean fluorescent intensity of CXCR2 positive neutrophils isolated from VDS and VDD mice (74.5±19.7 vs. 91.8±17.3 MFI, Figure 7E), suggesting similar surface expression of CXCR2 in CXCR2 positive cells. Together, these data suggest that vitamin D does not affect neutrophil chemotaxis to KC/CXCL1 through modulation of CXCR2 expression.

## Discussion

Our goal in this study was to investigate the role of vitamin D in LPS-induced acute lung injury and its resolution. We hypothesized that both the severity of lung injury and resolution of injury would be impaired in vitamin D deficient mice exposed to LPS. The primary findings of this study are that vitamin D level has minimal impact on acute inflammation and injury following LPS and on subsequent resolution of inflammation and injury. Minor differences of uncertain significance were observed in early leukocyte recruitment to the lung and on recovery of weight loss at day 10.

One day following LPS exposure, there were overall significant increases in lung elastance, left lung lobe weight, BAL fluid total protein concentration and BAL fluid IgM concentration consistent with disruption of the alveolar-capillary barrier. These changes were associated with increased leukocyte and neutrophil recruitment to the alveolar compartment and elevation in the CXC chemokines, KC and MIP2, and the cytokines IL6 and TNFα. However, there were no differences between VDS and VDD mice for any of the measured parameters with the exception that VDD mice had slightly fewer BAL fluid leukocytes. This was associated with a non-significant (p = 0.09) trend towards fewer neutrophils in the BAL fluid of VDD mice. Lung injury as measured by wet lung weight, elastance, and permeability was greatest three days following LPS exposure without any difference seen between VDS and VDD mice for any measured parameter, including BAL fluid leukocytes. By day 10, most measures of lung injury and inflammation were approaching that seen in the PBS-treated controls. Again, there was no difference between the VDS and VDD mice for any parameter other than slightly lower body weight change measurements in the VDS mice.

These data contrast with other published studies that demonstrate a possible protective role of vitamin D supplementation during lung injury. Using a rat model of radiation-induced pneumonitis, Yazici and co-authors examined the effect of vitamin D supplementation on lung injury and remodeling 8 and 12 weeks after thoracic irradiation [32]. No benefit of vitamin D supplementation was observed when lung injury was evaluated by light microscopy; however, some statistically significant differences were observed with transmission electron microscopy (TEM). Specifically, TEM identification of extravascular erythrocytes, fibroblast count, collagen bundles, perivascular mast cells, eosinophil count, and intra-alveolar debris were all lower 8 and 12 weeks after radiation injury in the rats given supplemental vitamin D. Neutrophil count was lower at 8 weeks and macrophage count was lower at 12 weeks in supplemented rats, as well. These data suggest that some protective benefit may be derived from vitamin D supplementation in the setting of radiation-induced lung injury; however, the potential physiological relevance of these differences is difficult to ascertain given the absence of systematic sampling or the lack of data on the magnitude of the differences. Takano and co-authors found that acute high dose 1α,25[OH]D_3_ supplementation in hamsters significantly attenuated neutrophil accumulation 24 hours following LPS [31]. The difference between this study and the current one may reflect effects of very high level acute supplementation at the time of injury as opposed to the effects of chronic deficiency or it may reflect species differences as hamsters produce IL-8 and mice do not. IL-8 production may be suppressed by vitamin D, depending on the cell type [39], [40]. Shih and co-authors reported that vitamin D supplementation reduced lung injury as determined by wet/dry lung weight ratios and semi-quantitative histological analysis in a rat model of hind limb ischemia and reperfusion [30]. Differences between this study and the current one may reflect differences in direct versus indirect lung injury. Interestingly, Shih reported that serum cytokine levels were reduced in this model with vitamin D supplementation. Alternatively, differences may again reflect the difference of supplementation above a normal level versus chronic vitamin D deficiency.

Because our data suggested a possible mild early defect in neutrophil recruitment in the VDD mice despite no differences in CXCL1/KC and CXCL2/MIP2 production in VDD and VDS mice, we evaluated the chemotactic potential of BAL fluid collected from VDS and VDD mice 1 day following LPS exposure. This approach assessed whether there were differences in the alveolar compartment of neutrophil chemoattractant(s) other than those measured, for example chemokines (e.g. CXCL5/Ena-78), complement factors, or leukotrienes. No difference was observed, suggesting that any differences in neutrophil recruitment between VDS and VDD mice were not due to differential production of a soluble chemoattractant. We next evaluated the chemotactic ability of neutrophils isolated from VDS and VDD mice towards varying gradients of two different neutrophil chemoattractants, KC/CXCL1 and complement C5a. A mild reduction in chemotaxis specific for KC/CXCL1 was observed. Because the various neutrophil chemoattractant receptors all signal via similar G protein-coupled receptor pathways, we assessed expression of CXCR2, the receptor for KC/CXCL1 but did not find any differences in expression on bone marrow-derived neutrophils from VDS and VDD mice. One possible explanation for altered KC/CXCL1 responsiveness in VDD mice in the absence of reduced expression of CXCR2 may be vitamin D-dependent post-translational modifications to CXCR2. Vitamin D-dependent regulation of protein post-translational modification has been reported for the extracellular matrix protein, osteopontin [41].

These data indicate that chronic vitamin D deficiency has minimal impact on the acute inflammatory response to LPS or in resolution of LPS-induced lung injury. These findings further suggest that acute vitamin D replacement therapy for patients with underlying vitamin D deficiency may have limited clinical benefit in the setting of acute lung injury from noninfectious causes. An important caveat to this conclusion is that we used the LPS-induced model of acute lung injury in this study as opposed to an infectious pneumonia model. We chose this model because we were primarily interested in how vitamin D level affected inflammatory response and resolution of inflammation rather than bacterial or viral clearance. Vitamin D is important for macrophage expression of the antimicrobial peptide, cathelicidin, following Toll-like receptor activation [19]. Epidemiological data suggest that vitamin D level is associated with severity of illness with community-acquired pneumonia [42], [43]. Therefore, while vitamin D may not play a role in the acute inflammatory response or resolution of inflammation, it may still be important in controlling infections in the lung. Whether vitamin D modulates neutrophil function with infectious pneumonia remains unknown.

In addition to our primary findings, we also found no difference in total lung capacity or lung elastance in PBS-treated VDS or VDD mice. In contrast, a recent study reported that BALB/c vitamin D deficient mice had smaller lung volumes than vitamin D sufficient mice as well as higher elastance [44]. The difference between our findings and these may reflect the different mouse strain used in our study. Additionally, in the prior study, the breeders were vitamin D deficient; whereas, ours were only placed in a vitamin deficient environment once they were already visibly pregnant. The differences that the other group saw may be more the result of maternal and early gestational vitamin D deficiency and its effects on fetal lung development. This is supported by human data suggesting that maternal vitamin D may affect fetal lung development [28].

A limitation of this study is that normal levels of vitamin D in mice are not well established. Therefore, an unanswered question is if vitamin D augmentation can be protective in our LPS model for ALI. The relevance of 25[OH]D_3_ in mice is not well understood. Mice do utilize UV exposure to produce vitamin D_3_ [45], but it is possible that vitamin D may be more relevant and active in humans.

In summary, vitamin D deficiency, established through dietary restriction, has minimal impact on the acute inflammatory response to LPS in the lung or resolution of inflammation. Minor differences were noted in early leukocyte recruitment to the lung as well as weight recovery by day 10. This was associated with a mild impairment of neutrophil chemotaxis towards KC *ex vivo*. However, the impact of these differences on overall clinical relevance of vitamin D deficiency is likely minimal. The data suggest that vitamin D supplementation is unlikely to be an effective therapy for acute lung injury.
